# 超高效液相色谱-串联质谱法测定猪尿中12种禁用兽药残留

**DOI:** 10.3724/SP.J.1123.2023.09020

**Published:** 2024-05-08

**Authors:** Jianchun WAN, Ying HAN, Xinxin MA, Shixiang LI, Huawen WU, Lihua JI, Zhiwei DENG, Chunrui ZHAN

**Affiliations:** 南昌海关技术中心, 江西 南昌 330038; Technology Center of Nanchang Customs District, Nanchang 330038, China

**Keywords:** 超高效液相色谱-串联质谱, 酶解, 酸解, 衍生化, 禁用兽药, 猪尿, ultra high performance liquid chromatography-tandem mass spectrometry (UHPLC-MS/MS), enzymatic hydrolysis, acidolysis, derivatization, prohibited veterinary drugs, pig urine

## Abstract

研究建立了超高效液相色谱-串联质谱测定猪尿中*β*_2_-受体激动剂类、硝基呋喃代谢物类、硝基咪唑类、氯丙嗪、氯霉素等5类12种禁用兽药残留量同时检测的方法。试样中加入4.5 mL 0.2 mol/L乙酸铵溶液和40 μL *β*-葡萄糖醛酸酶/芳基硫酸酯酶,37 ℃酶解2 h后,再加入1.5 mL 1.0 mol/L盐酸溶液和100 μL 0.1 mol/L邻硝基苯甲醛溶液,经37 ℃衍生16 h,用8 mL乙酸乙酯萃取,下层水相用混合型阳离子交换固相萃取柱萃取,合并2次萃取液,氮气吹干复溶,正负离子多反应监测模式下检测,同位素内标法定量。12种化合物在各自范围内线性关系良好,相关系数(*r*)>0.99;氯霉素的检出限为0.05 μg/L,定量限为0.1 μg/L,其他化合物的检出限为0.25 μg/L,定量限为0.5 μg/L;在定量限1倍、2倍、10倍添加水平下的平均回收率为83.6%~115.3%, RSD为2.20%~12.34%。方法灵敏度高,稳定性好,定量准确,适用于猪尿中12种禁用兽药残留量的同时测定。

## Abstract (en)

A method was established for the simultaneous detection of 12 prohibited veterinary drugs, including *β*_2_-receptor agonists, nitrofuran metabolites, nitroimidazoles, chlorpromazine, and chloramphenicol, in pig urine. The sample was pretreated by enzymolysis, acid hydrolysis/derivatization, and liquid-liquid extraction combined with solid-phase extraction. Detection was performed using ultra high performance liquid chromatography-tandem mass spectrometry (UHPLC-MS/MS). Ammonium acetate solution (0.2 mol/L, 4.5 mL) and *β*-glucuronidase/aryl sulfatase (40 μL) were added to the sample, which was subsequently enzymolized at 37 ℃ for 2 h. Then, 1.5 mL of 1.0 mol/L hydrochloric acid solution and 100 μL of 0.1 mol/L *o*-nitrobenzaldehyde solution were added to the sample. The mixture was incubated at 37 ℃ for 16 h, and the analytes were extracted with 8 mL of ethyl acetate by liquid-liquid extraction. The lower aqueous phase obtained after extraction was extracted and purified using a mixed cation-exchange solid-phase extraction column. The extracts were combined, the extraction solution was blow-dried with nitrogen, and the residue was redissolved for determination. The samples were analyzed under multiple-reaction monitoring mode with both positive and negative electrospray ionization, and quantified using an isotope internal standard method. The correlation coefficients (*r*) of the 12 compounds were >0.99. The limits of detection (LODs) and quantification (LOQs) of chloramphenicol were 0.05 and 0.1 μg/L, respectively, and the LODs and LOQs of the other compounds were 0.25 and 0.5 μg/L, respectively. The mean recoveries and RSDs at 1, 2, and 10 times the LOQ were 83.6%-115.3% and 2.20%-12.34%, respectively. The proposed method has the advantages of high sensitivity, good stability, and accurate quantification; thus, it is suitable for the simultaneous determination of the 12 prohibited veterinary drug residues in pig urine.

随着规模化养殖产业的发展,动物养殖使用兽药较为普遍,有关部门加强产品流通环节前的监测,可避免不合格动物性食品流入市场,即保证了食品的安全,也能避免养殖企业更大的损失,这是监管前置的重要技术保障措施。不同靶组织中兽药的形态差异以及转化产物等是影响兽药残留检测的重要因素,这也增加了多类别兽药残留检测技术开发的难度,所以单一兽药或同类兽药残留的检测方法较常见^[1⇓⇓⇓⇓⇓-7]^。针对多类别兽药残留检测,用相对简单的样品处理方式,再经选择性更高的高分辨质谱分析检测,可弥补样品前处理净化的不足^[8⇓⇓-11]^。

尿样中的兽药残留检测一直受到关注,如龚波等^[12]^采用乙酸乙酯萃取,阳离子交换固相萃取柱净化,建立了液相色谱-串联质谱法测定猪尿中7种*α*_2_-受体激动剂残留量的方法;张婧等^[8]^用流动相直接稀释猪尿样品,超高效液相色谱-四极杆-静电场轨道阱高分辨质谱测定,建立了猪尿中74种抗生素类药物残留的检测方法,上述方法主要针对同一类别兽药的检测,或是利用高分辨质谱的高选择性,简化样品前处理步骤,实现不同类别兽药的检测;李振环等^[13]^采用酶解处理和固相萃取柱净化,建立了人体尿液中大环内酯、四环素、喹诺酮、磺胺及*β*_2_-受体激动剂类药物残留的液相色谱-串联质谱的检测方法,该方法实现了*β*_2_-受体激动剂类药物与其他非酶解药物的同时检测,但需酶解、衍生化处理的兽药残留同时检测未有相关研究报道。

Chen等^[14]^利用液相色谱-三重四极杆质谱分析技术,样品先酸解衍生后,再分别用乙酸乙酯、乙腈液液萃取,建立了水产品中硝基呋喃代谢物、硝基咪唑类、孔雀石绿、氯霉素同时测定的方法;陈兴连等^[15]^则建立了水产品中硝基呋喃代谢物、孔雀石绿、氯霉素、磺胺类、喹诺酮类药物残留的检测方法,这些研究证明了硝基呋喃代谢物可以与其他兽药同时分析检测,但酸解衍生对于其他兽药测定的影响暂未探讨。

本文采用超高效液相色谱-串联质谱(UHPLC-MS/MS)检测技术,同时测定了猪尿中硝基呋喃代谢物与其他类别兽药(包括*β*_2_-受体激动剂、硝基咪唑、氯丙嗪、氯霉素),并对酶解、酸解衍生对目标物的影响进行了探讨,为多类别兽药残留检测提供参考借鉴。

## 1 实验部分

### 1.1 仪器、试剂与材料

ExionLC液相色谱仪、API 5500型三重四极杆质谱仪(美国AB SCIEX公司); SHZ-C水浴恒温振荡器(上海博迅医疗生物仪器股份有限公司); TDL-5-A离心机(上海安亭科学仪器厂); N-EVAP112氮吹浓缩仪(美国Organomation公司); KQ-600B型超声波清洗器(昆山市超声仪器有限公司); Option-Q15型超纯水一体机(英国ELGA公司)。

色谱纯甲醇、乙腈、乙酸乙酯、乙酸铵、乙酸、甲酸、2-邻硝基苯甲醛(2-NPA,纯度≥98%),*β*-葡萄糖醛苷酸酶/芳基硫酸酯酶(酶活力单位大于30 U/mL),均购于上海安谱公司;盐酸、氨水(分析纯,上海国药集团)。呋喃它酮的代谢物5-吗啉甲基-3-氨基-2-噁唑烷基酮(AMOZ)、呋喃唑酮的代谢物3-氨基-2-噁唑烷基酮(AOZ)、呋喃妥因的代谢物1-氨基-2-内酰脲(AHD)、呋喃西林的代谢物氨基脲(SEM)、克伦特罗、沙丁胺醇、莱克多巴胺、甲硝唑、地美硝唑、洛硝哒唑、氯丙嗪、氯霉素及对应同位素内标物(见表1),纯度≥98%(德国Dr. Ehrenstorfer公司)。混合型阳离子交换固相萃取小柱(MCX柱,150 mg, 6 mL,美国Waters公司)。

**表1:** 12种兽药及其同位素内标的质谱参数

Compound	Parent ion (m/z)	Daughter ions (m/z)	Declustering potential/V	Collision energies/eV
2-NP-AMOZ (5-吗啉甲基-3-氨基-2-噁唑烷基酮衍生物)	335.2	291.1^*^, 262.2	80	17, 23
2-NP-AOZ (3-氨基-2-噁唑烷基酮衍生物)	236.1	133.9^*^, 103.9	80	17, 31
2-NP-AHD (1-氨基-2-内酰脲衍生物)	249.2	134.1^*^, 104.1	80	17, 27
2-NP-SEM (氨基脲衍生物)	209.2	162.2^*^, 192.1	80	14, 16
Clenbuterol (克伦特罗)	277.0	203.0^*^, 168.1	65	21, 38
Salbutamol (沙丁胺醇)	240.2	148.1^*^, 222.3	68	24, 14
Ractopamine (莱克多巴胺)	302.0	164.1^*^, 284.0	80	23, 18
Metronidazole (甲硝唑)	172.2	127.9^*^, 82.0	50	20, 37
Dimetridazole (地美硝唑)	142.2	96.0^*^, 81.0	65	21, 36
Ronidazole (洛硝哒唑)	201.2	140.0^*^, 55.0	50	15, 27
Chlorpromazine (氯丙嗪)	319.1	58.2^*^, 86.2	60	66, 30
Chloramphenicol (氯霉素)	321.0	256.9^*^, 152.1	-75	-24, -17
2-NP-AMOZ-D_5_(5-吗啉甲基-3-氨基-2-噁唑烷基酮衍生物-D_5_)	340.2	296.2^*^	80	16
2-NP-AOZ-D_4_(3-氨基-2-噁唑烷基酮衍生物-D_4_)	240.1	134.1^*^	80	19
2-NP-AHD-^13^C_3_(1-氨基-2-内酰脲衍生物-^13^C_3_)	252.1	134.1^*^	80	18
2-NP-SEM-^13^C^15^N_2_(氨基脲衍生物-^13^C^15^N_2_)	212.1	168.1^*^	80	16
Clenbuterol-D_9_(克伦特罗-D_9_)	286.1	204.0^*^	65	24
Salbutamol-D_3_(沙丁胺醇-D_3_)	243.2	151.1^*^	68	25
Ractopamine-D_3_(莱克多巴胺-D_3_)	305.1	167.2^*^	80	23
Metronidazole-^13^N_2_(甲硝唑-^13^N_2_)	176.1	132.1^*^	50	21
Dimetridazole-D_3_(地美硝唑-D_3_)	145.0	99.1^*^	65	25
Ronidazole-D_3_(洛硝哒唑-D_3_)	204.0	143.1^*^	50	20
Chlorpromazine-D_6_(氯丙嗪-D_6_)	325.1	64.2^*^	60	67
Chloramphenicol-D_5_(氯霉素-D_5_)	326.2	157.2^*^	-75	-26

2-NP-AMOZ: 5-(4-morpholinylmethyl)-3-[(*E*)-(2-nitrobenzyliden)amino]-1,3-oxazolidin-2-on; 2-NP-AHD: 3-[(*E*)-(2-nitrobenzyliden)amino]-2,4-imidazolidinedione; 2-NP-AOZ: 3-[(*E*)-(2-nitrobenzyliden)amino]-1,3-oxazolidin-2-on; 2-NP-SEM: (2*E*)-2-(2-nitrobenzyliden)hydrazincarboxamid; * quantitative ion.

### 1.2 溶液配制

0.2 mol/L乙酸铵缓冲溶液:称取乙酸铵7.70 g,加入450 mL水溶解后,用乙酸调节pH至5.2,定容至500 mL; 0.1 mol/L邻硝基苯甲醛溶液:称取1.5 g邻硝基苯甲醛,用甲醇溶解,定容至100 mL; 5%氨化甲醇:移取5 mL氨水至95 mL甲醇中,混匀。

标准溶液:分别准确称取12种标准物质10 mg,用甲醇溶解并定容至10 mL容量瓶中,配制成质量浓度为1.0 mg/mL的标准储备溶液,使用前用甲醇稀释至适当浓度。各化合物对应的同位素内标采用同样方式配制成质量浓度为1.0 mg/mL的内标储备溶液,使用前用甲醇配制为内标混合溶液(氯霉素-D_5_质量浓度为20 ng/mL,其他内标质量浓度为100 ng/mL)。

### 1.3 样品前处理

吸取试样2 mL置于50 mL聚丙烯离心管1内,加入50 μL内标混合溶液,再加入4.5 mL 0.2 mol/L乙酸铵溶液和40 μL *β*-葡萄糖醛酸酶/芳基硫酸酯酶,涡旋混匀,于37 ℃水浴振荡酶解2 h后,加入1.5 mL 1 mol/L盐酸溶液和0.1 mol/L邻硝基苯甲醛溶液100 μL,涡旋混匀,于37 ℃水浴振荡衍生16 h后,加入8 mL乙酸乙酯,涡旋10 s,以5000 r/min离心5 min,取上层乙酸乙酯至离心管2内,下层水相转入经5 mL甲醇和6 mL水活化、平衡后的MCX小柱内,分别用6 mL水和6 mL甲醇淋洗小柱,空气吹干小柱,用6 mL 5%氨化甲醇洗脱目标物,收集全部洗脱液至离心管2内,在45 ℃水浴下氮气吹至干,残渣用1 mL 5%乙腈水溶液超声复溶,过0.22 μm滤膜后上机测定。

### 1.4 仪器条件

色谱柱:Titank C_18_(100 mm×2.1 mm, 3.0 μm,广州菲罗门科学仪器有限公司);流动相A:乙腈,流动相B: 0.1%甲酸水溶液;梯度洗脱:0~2.5 min, 2%A~5%A; 2.5~3.0 min, 5%A~15%A; 3.0~5.0 min, 15%A~35%A; 5.0~7.0 min, 35%A~55%A; 7.0~9.0 min, 55%A~95%A; 9.0~11.0 min, 95%A; 11.0~11.01 min, 95%A~2%A; 11.01~14.0 min, 2%A。流速:0.3 mL/min;进样体积:5 μL;柱温:40 ℃。

离子源:ESI源,正、负离子扫描模式;多反应监测(MRM)模式;雾化气压力(GS1): 275.8 kPa;辅助加热气压力(GS2): 275.8 kPa;气帘气压力(CUR): 275.8 kPa;离子源温度:550 ℃;电喷雾电压(IS):5500 V和-4500 V。其他质谱参数见表1。

## 2 结果与讨论

### 2.1 仪器条件的优化

采用针泵进样的方式,分别对各个目标物的质谱参数进行优化。一级母离子扫描确定目标物母离子,二级子离子扫描获得各个目标物的二级碎片离子,以质谱丰度和*m/z*抗干扰性为原则,选择目标物的定性定量离子。采用多反应监测模式,优化各目标物离子对的碰撞能量(CE)和去簇电压(DP)。方法拟采用正、负离子模式同时扫描的方式检测,实验用乙腈-0.1%甲酸溶液(1∶1, v/v)为流动相,通过三通与针泵连接,调节离子源参数使目标物响应最高,以获得最佳离子源参数。正、负离子同时扫描模式的切换速度较快,除电喷雾电压外,切换离子源温度和气流参数时会影响响应值,因此正、负扫描模式下采用相同的离子源温度和气流参数。

对比了Titank C_18_(100 mm×2.1 mm, 3.0 μm)和ACQUITY UPLC BEH C_18_(100 mm×2.1 mm, 1.7 μm,)色谱柱的分离效果,两款色谱柱均能实现12种化合物的有效分离,Titank C_18_色谱柱柱压较低,耐用性更好。硝基咪唑类药物和沙丁胺醇的极性相对较大,流动相中乙腈初始比例越高,其出峰时间越早,流动相中乙腈初始比例为5%时,甲硝唑等药物出峰较快,在基质样液中存在干扰,乙腈初始比例降至2%时,甲硝唑、地美硝唑、洛硝哒唑、沙丁胺醇色谱保留时间都较理想,峰形尖锐,基质共流出物的干扰得到明显改善。12种化合物的提取离子色谱图见图1。

**图1:**
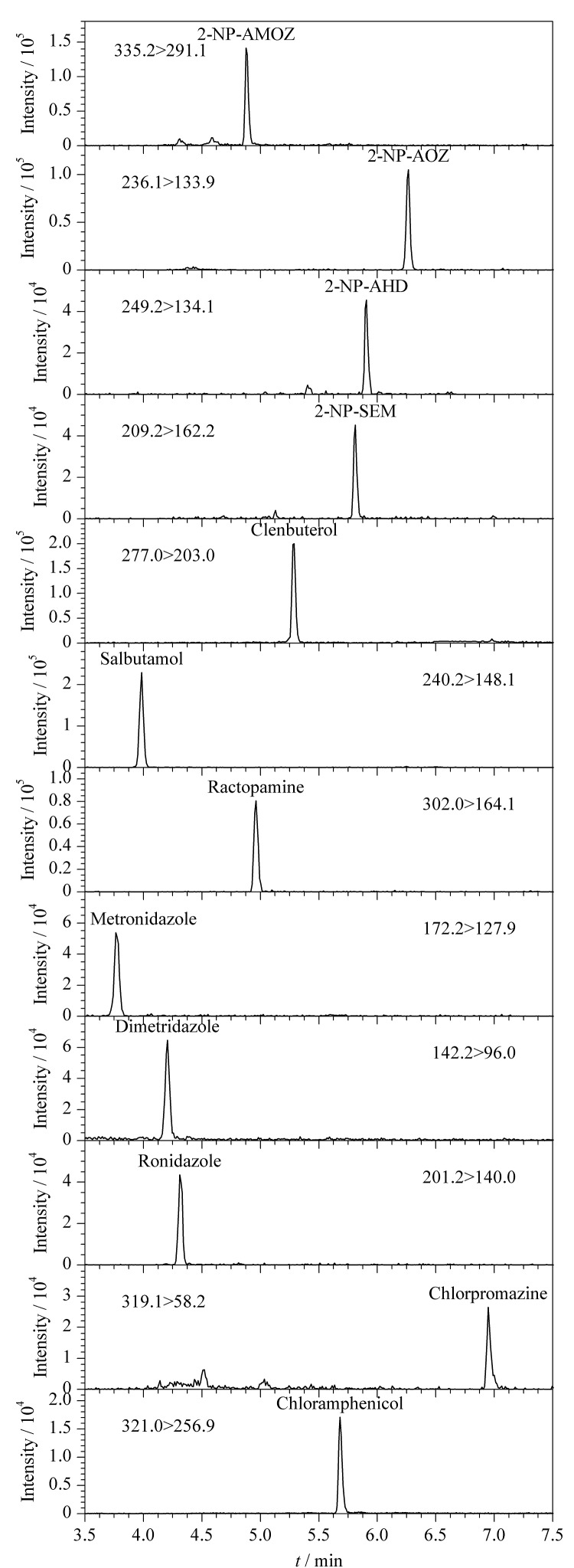
12种化合物的提取离子色谱图

### 2.2 衍生化反应的影响

硝基呋喃代谢物的质谱检测,衍生试剂为邻硝基苯甲醛^[16]^,为考察邻硝基苯甲醛的衍生反应是否会影响其他化合物,进行如下实验:分别取质量浓度为1 μg/mL的12种混合标准溶液(其中氯霉素为0.1 μg/mL)各1 mL,分别加入9 mL水(Test 1)、0.5 mL甲酸和8.5 mL水(Test 2)、0.5 mL甲酸、8.4 mL水和0.1 mol/L邻硝基苯甲醛溶液0.1 mL(Test 3),于37 ℃水浴振荡衍生16 h,分别取0.1 mL反应后溶液,用水稀释至1 mL上机测定,测定结果如图2所示。Test 3中4种硝基呋喃代谢物形成了衍生产物,而其他化合物3种方式处理后的响应值均无显著差异,说明除硝基呋喃代谢物外,其他8种化合物不会与邻硝基苯甲醛发生衍生反应。

**图2:**
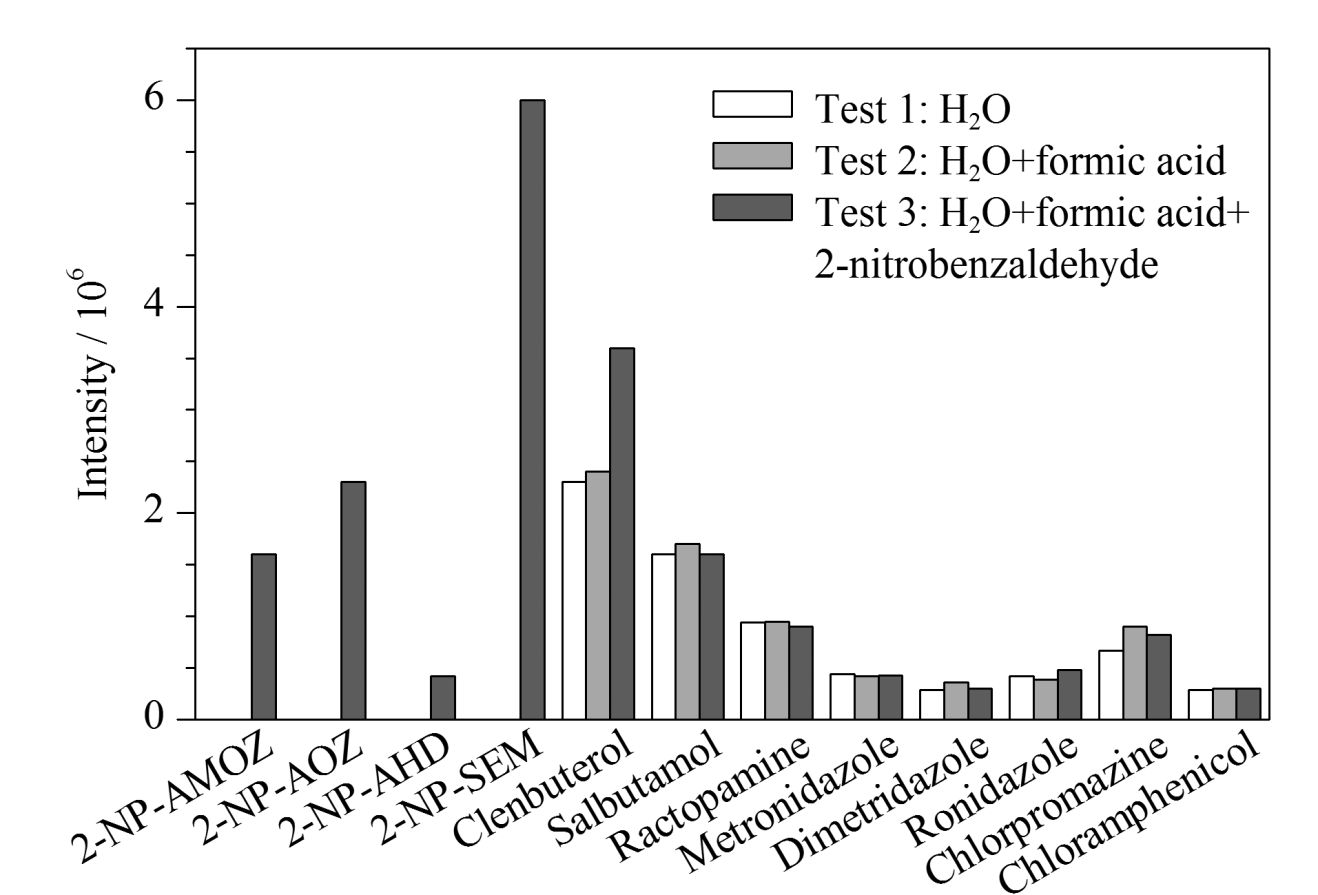
邻硝基苯甲醛衍生反应对各化合物响应值的影响

### 2.3 样品酶解和衍生条件的确定

动物组织中*β*_2_-受体激动剂类药物残留检测多采用酶解方式处理^[2,13]^,也有采用酸性条件下直接提取和调节pH值后直接测定的研究报道^[7,17,18]^。实验探讨了在酶解条件下实现硝基呋喃代谢物衍生的可行性。移取2 mL水,加入10 μL质量浓度为1 μg/mL的12种混合标准溶液(其中氯霉素为0.1 μg/mL),制备成试样;酶解衍生对照组在试样中加入0.2 mol/L乙酸铵缓冲溶液8 mL、40 μL *β*-葡萄糖醛酸酶/芳基硫酸酯酶、100 μL邻硝基苯甲醛;酸解衍生对照组则在试样中加入0.2 mol/L盐酸5 mL、100 μL邻硝基苯甲醛,两组对照试验组均在37 ℃水浴振荡衍生反应16 h,衍生反应后调节pH值至中性,采用乙腈和乙酸乙酯各7.5 mL复合溶剂体系盐析萃取,上机测定结果如图3所示。

**图3:**
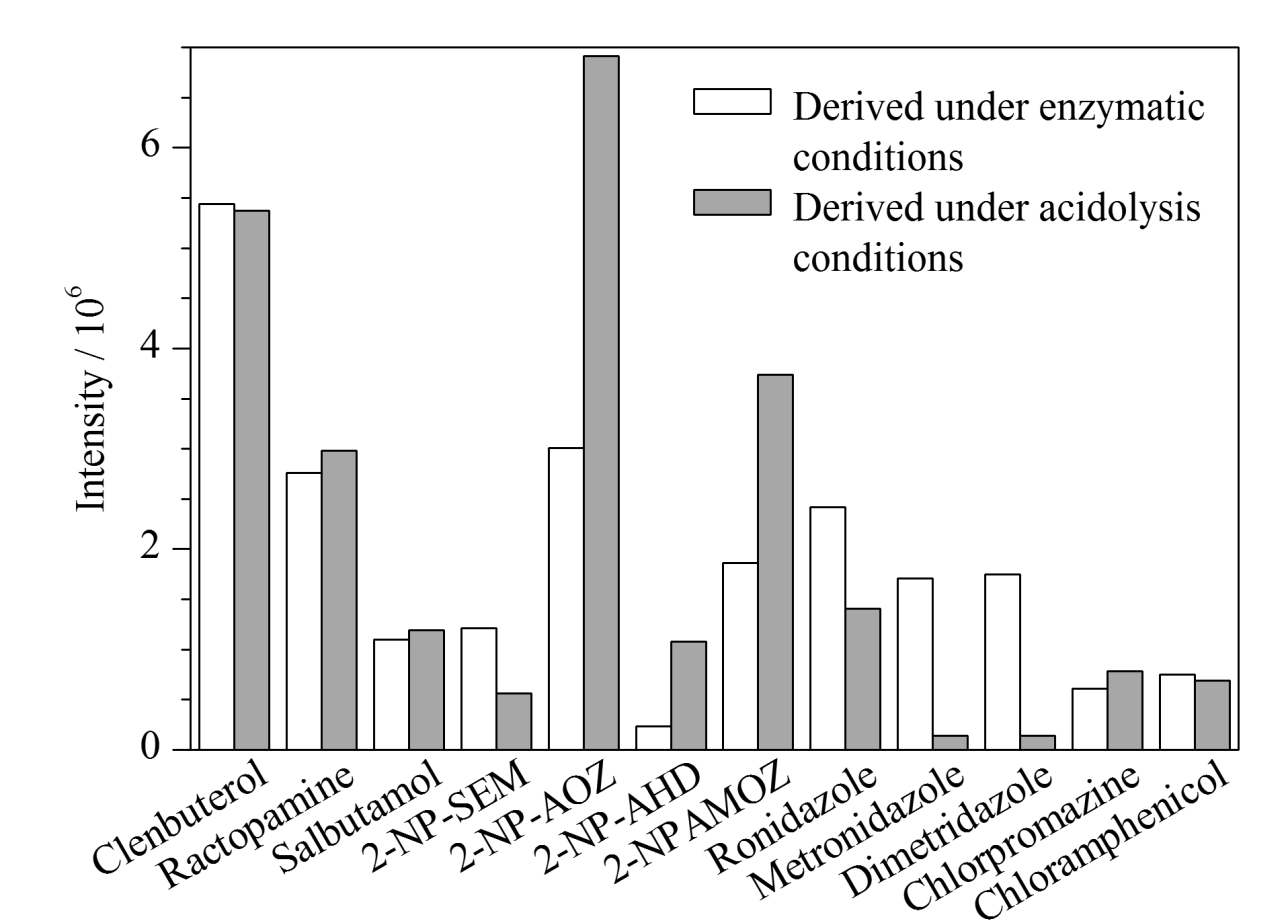
酶解衍生和酸解衍生的效果对比

需衍生化处理的硝基呋喃代谢物2-NP-AOZ、2-NP-AHD、2-NP-AMOZ经酸解衍生处理后,响应值明显要高于酶解衍生,酶解衍生条件下甲硝唑、地美硝唑、洛硝哒唑响应值则更高,*β*_2_-受体激动剂和其他化合物2种方式处理无明显差异。*β*_2_-受体激动剂采用酶解是检测标准^[19]^采用的方式,本研究团队曾研究猪尿中*β*_2_-受体激动剂类药物残留采用酶解和酸解的效果差异,结果表明酶解有助于*β*_2_-受体激动剂类药物充分提取,该类药物酶解2 h的酶解效率达到67.9%~91.3%^[20]^。因此本方法采用酶解后再酸解衍生的方式。

### 2.4 萃取净化条件的优化

硝基呋喃代谢物与邻硝基苯甲醛衍生反应在较强酸性下响应值更高,实验证明0.2 mol/L乙酸铵缓冲溶液(pH 5.2)酶解后,加入1 mol/L盐酸1.5 mL,试样溶液的pH值为1.29~1.31,与相关检测标准^[21]^直接盐酸酸解衍生的pH值相当。对酶解和酸解衍生后的试样溶液进行3种萃取净化处理:调节pH值至中性,加入氯化钠,用乙腈盐析萃取;调节pH值至中性,用乙酸乙酯液液萃取;试样溶液用乙酸乙酯液液萃取,下层水相过混合型阳离子交换柱净化,合并液液萃取和固相萃取的萃取液。

如图4所示,对比各组萃取方式下药物的响应值,发现乙腈盐析萃取,标准溶液中各药物响应值较高,但在猪尿基质加标样品中的响应值则明显要低于另2种萃取方式,可能是乙腈盐析萃取的干扰物较多,基质抑制作用增强;乙酸乙酯萃取下沙丁胺醇的响应值最低,可能是乙酸乙酸对于极性较大的药物萃取效率较低,但对于猪尿基质加标样品,乙酸乙酯萃取比乙腈盐析萃取的响应值要高,这也说明乙酸乙酯萃取物相对较少,萃取引入的基质干扰相对较少;采用乙酸乙酯液液萃取组合MCX小柱固相萃取的方式,各药物在标准溶液和基质加标样品中的响应值均较高,乙酸乙酯萃取一部分非离子态药物,MCX小柱萃取离子态药物,即保证了萃取效率,又能降低基质干扰,因此方法选择了乙酸乙酯萃取组合MCX小柱固相萃取的方式。

**图4:**
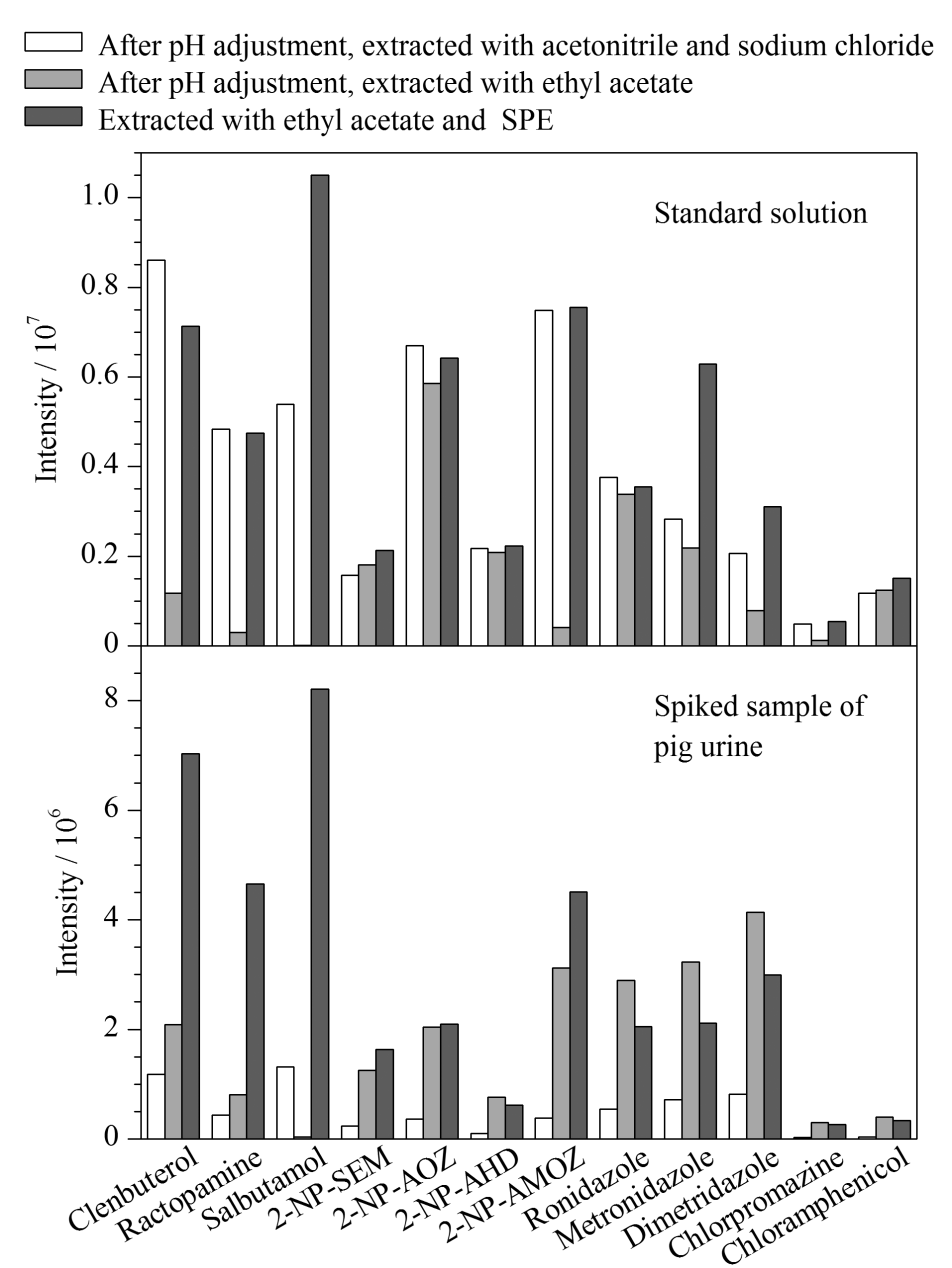
不同提取净化方式下的提取效率对比

### 2.5 方法学评价

#### 2.5.1 相关系数、检出限和定量限

基质效应对定量结果存在影响,为消除基质效应的影响,本方法采用同位素内标法定量,氯霉素在0.1~4.0 ng/mL,其他化合物在0.5~20.0 ng/mL范围内均呈良好的线性相关,相关系数(*r*)均在0.99以上。

采用空白猪尿加标的方式,按1.3节方法处理后,在1.4节条件下进行检测。根据各化合物的特征色谱峰的信噪比(*S/N*)=3和10时对应的加标水平,确定检出限(LOD)和定量限(LOQ),氯霉素的LOD为0.05 μg/L,LOQ为0.1 μg/L,其他化合物的LOD为0.25 μg/L,LOQ为0.5 μg/L。方法的定量限优于同类药物残留检测方法^[8,19,21⇓-23]^。

#### 2.5.2 回收率

以空白猪尿为基质样品,在1倍、2倍、10倍LOQ水平下进行加标回收试验,12种化合物的平均回收率为83.6%~115.3%, RSD为2.20%~12.34%(见表2),符合GB/T 27417-2017的规定。

**表2:** 12种化合物在猪尿中的平均回收率和相对标准偏差(*n*=6)

Compound	Spiked/(μg/L)	Recovery/%	RSD/%
2-NP-AMOZ	0.5	98.6	9.85
	1.0	96.1	7.81
	5.0	96.8	7.16
2-NP-AOZ	0.5	100.5	5.88
	1.0	96.7	5.53
	5.0	92.2	12.34
2-NP-AHD	0.5	93.9	9.06
	1.0	100.1	5.54
	5.0	100.8	3.97
2-NP-SEM	0.5	106.8	4.28
	1.0	103.5	5.65
	5.0	94.3	4.42
Clenbuterol	0.5	92.5	8.88
	1.0	91.8	4.52
	5.0	92.7	4.55
Salbutamol	0.5	91.5	3.35
	1.0	88.1	4.75
	5.0	83.6	4.27
Ractopamine	0.5	91.8	5.33
	1.0	86.2	3.55
	5.0	91.4	4.21
Metronidazole	0.5	89.9	6.15
	1.0	93.2	10.06
	5.0	91.7	7.54
Dimetridazole	0.5	98.5	8.52
	1.0	100.1	3.97
	5.0	99.3	8.41
Ronidazole	0.5	91.4	6.79
	1.0	99.7	7.11
	5.0	96.6	7.82
Chlorpromazine	0.5	115.3	3.12
	1.0	99.9	9.42
	5.0	97.8	8.34
Chloramphenicol	0.1	106.9	3.42
	0.2	104.4	2.54
	1.0	103.3	2.20

### 2.6 实际样品的测定

按本实验建立的方法对32批次供港活猪尿样进行检测,均未检出上述12种禁用药物。

## 3 结论

采用酶解、酸解衍生、液液萃取结合固相萃取,建立了猪尿中*β*_2_-受体激动剂、硝基呋喃代谢物、硝基咪唑类、氯丙嗪、氯毒素等5类12种药物同时检测的方法。本文讨论了酶解、酸解衍生处理对目标物的影响,对比优化了前处理技术,可为多类别兽药残留检测技术研究提供参考。
